# Multi-omics-data-assisted genomic feature markers preselection improves the accuracy of genomic prediction

**DOI:** 10.1186/s40104-020-00515-5

**Published:** 2020-12-01

**Authors:** Shaopan Ye, Jiaqi Li, Zhe Zhang

**Affiliations:** Guangdong Provincial Key Lab of Agro-Animal Genomics and Molecular Breeding, National Engineering Research Centre for Breeding Swine Industry, College of Animal Science, South China Agricultural University, Guangzhou, Guangdong China

**Keywords:** Accuracy, *Drosophila melanogaster*, Genomic prediction, Multi-omics data, SNP preselection

## Abstract

**Background:**

Presently, multi-omics data (e.g., genomics, transcriptomics, proteomics, and metabolomics) are available to improve genomic predictors. Omics data not only offers new data layers for genomic prediction but also provides a bridge between organismal phenotypes and genome variation that cannot be readily captured at the genome sequence level. Therefore, using multi-omics data to select feature markers is a feasible strategy to improve the accuracy of genomic prediction. In this study, simultaneously using whole-genome sequencing (WGS) and gene expression level data, four strategies for single-nucleotide polymorphism (SNP) preselection were investigated for genomic predictions in the *Drosophila* Genetic Reference Panel.

**Results:**

Using genomic best linear unbiased prediction (GBLUP) with complete WGS data, the prediction accuracies were 0.208 ± 0.020 (0.181 ± 0.022) for the startle response and 0.272 ± 0.017 (0.307 ± 0.015) for starvation resistance in the female (male) lines. Compared with GBLUP using complete WGS data, both GBLUP and the genomic feature BLUP (GFBLUP) did not improve the prediction accuracy using SNPs preselected from complete WGS data based on the results of genome-wide association studies (GWASs) or transcriptome-wide association studies (TWASs). Furthermore, by using SNPs preselected from the WGS data based on the results of the expression quantitative trait locus (eQTL) mapping of all genes, only the startle response had greater accuracy than GBLUP with the complete WGS data. The best accuracy values in the female and male lines were 0.243 ± 0.020 and 0.220 ± 0.022, respectively. Importantly, by using SNPs preselected based on the results of the eQTL mapping of significant genes from TWAS, both GBLUP and GFBLUP resulted in great accuracy and small bias of genomic prediction. Compared with the GBLUP using complete WGS data, the best accuracy values represented increases of 60.66% and 39.09% for the starvation resistance and 27.40% and 35.36% for startle response in the female and male lines, respectively.

**Conclusions:**

Overall, multi-omics data can assist genomic feature preselection and improve the performance of genomic prediction. The new knowledge gained from this study will enrich the use of multi-omics in genomic prediction.

## Introduction

Genomic prediction, also known as genomic selection (GS), was initially proposed in 2001 [1] and is a statistical method to predict the yet-to-be observed phenotypes or unobserved genetic values of complex traits based on genomic data. This method assumes that all quantitative trait loci (QTLs) are in linkage disequilibrium (LD) with at least one marker in the whole genome. GS is known for shortening the generation intervals and increasing the reliability of predicted breeding values, especially for dairy cattle breeding [2]. Presently, genomic prediction is widely used in animal and plant breeding and polygenic disease risk prediction.

Over the past decade, the implementation of GS was mainly based on single-nucleotide polymorphism (SNP) chip data. With the cost of sequencing dropping rapidly, it became possible to perform genomic predictions with whole-genome sequencing (WGS) data. Compared with SNP chip data, WGS data are expected to improve the accuracy of genomic predictions by increasing the degree of LD between the SNPs and QTLs, even including causal mutations. Simulation studies confirmed the hypothesis that WGS data would improve the accuracy of genomic prediction in a single population [3] or multiple populations [4]. However, higher accuracy of genomic prediction was not achieved for *Drosophila* using real WGS data [5], and similar results were found for livestock using imputed WGS data [6–8]. Possibly, large amounts of markers are both non-causal markers and not in LD with the causal loci. Moreover, our previous study indicated that the LD pruning of imputed WGS data could improve prediction accuracy [8]. Therefore, pre-selected potential causal markers or QTLs from WGS has great potential for improving the accuracy of genomic prediction [9]. Nowadays, many preselection variant strategies were used to improve the power of genomic prediction based on the following methods: genome-wide association study (GWAS) [8, 10–12], Bayesian procedures [13], genome-wide signatures of selection [14], QTL regions in Animal QTLdb [12], gene annotation [15, 16], and gene ontology categories [17, 18]. These methods mainly depend on the direct link between phenotype and DNA variants or some prior genome annotation information. However, the genetic links between phenotype and genome variants are too complex to determine directly at the genome sequencing level.

Presently, it has become possible to obtain multi-omics data (e.g., genomic, transcriptomics, proteomics, and metabolomics) for genomic predictions. This makes it possible to uncover genotype–phenotype relationships using different types of data. Related studies were reported using omics data to perform genomic prediction for complex traits in humans [19, 20], plants [21–24], and model animals [25, 26]. Most of these studies focused on integrating multiple omics data into a prediction model to improve prediction accuracy [22, 25–27]. However, multi-omics data not only offers new data layers for genomic prediction but also provides a bridge between organismal phenotype and genome variation that cannot be readily captured at the genome sequence level [21]. Therefore, using omics data to select feature markers is a feasible strategy to improve the accuracy of genomic prediction.

In this study, using WGS and gene expression level data, different strategies of SNP preselection were investigated for genomic predictions in the *Drosophila* genetic reference panel (DGRP). Our results provide useful knowledge about preselected genomic features based on multi-omics data and thus improve the predictive ability of genomic predictions for complex traits.

## Materials and methods

### The genomic, transcriptomic, and phenotypic data of DGRP lines

The DGRP is a living library of common polymorphisms affecting complex traits, as well as a community resource for the whole genome association mapping of quantitative trait loci [28, 29]. The DGRP has 205 *Drosophila* inbred lines derived from 20 generations of full-sib mating from isofemale lines collected at the Farmer’s Market in Raleigh, NC, USA. These 205 lines were subjected to whole genome sequencing using Illumina and 454 sequencing. After variant calling, a total of 4,672,297 SNPs were found around the chromosome arm (X, 2L, 2R, 3L, 3R, 4) [28]. The gene expression level of 200 DGRP lines (as the log_2_-transformed fragments per kilobase of transcript per million fragments mapped, FPKM) for 15,732 genes in females and 20,375 genes in males were obtained by Everett et al. [30] and can be found in GEO (accession GSE117850). Furthermore, two traits (startle response and starvation resistance) were selected as model traits. Finally, totals of 198 and 199 lines selected for starvation resistance and startle response, respectively, were used for further genomic prediction due to allowing the measurement of phenotypes and expression levels simultaneously. In addition,the phenotypic value of Startle response and starvation resistance per line were the averages of two replicate measurements (20 flies/sex/replicate) and five replicate measurements (10 flies/sex/replicate), respectively [28]. The quality control of the WGS data was conducted using PLINK [31] with the criteria of SNP call rate ≥ 95%, individual call rate ≥ 97%, MAF ≥ 5%, and the Hardy–Weinberg equilibrium *P*-value ≥ 1.0e-6. The missing genotypes were imputed by Beagle 4.1 with default parameters [32]. Ultimately, a total of 2,037,712 SNPs was used for further analysis.

### Genetic parameter estimations

Before performing genomic prediction, in order to assess how much phenotypic variability could be explained by the genetic variation of the WGS data, the variance components (additive genetic and residual variance) of the startle response and starvation resistance were estimated in the male and female lines, respectively, by the information restricted maximum likelihood (REML) method implemented in the LDAK software [33]. The statistical model was

$$ \mathbf{y}=\boldsymbol{X}\mathbf{b}+\boldsymbol{Z}\mathbf{g}+\mathbf{e}, $$

where y is a vector of the phenotypic values of all lines; **b** is the Wolbachia infection status as a fixed effect; ***X*** and ***Z*** are the incidence matrices relating the fixed and polygene effects to the phenotypic records; **g** is a vector of the polygene effect of all individuals, which is assumed to be distributed as $\mathbf{g}\sim \mathbf{N}\left(\mathbf{0},{\boldsymbol{\upsigma}}_{\mathbf{g}}^{\mathbf{2}}\mathbf{G}\right)$; and **e** is the residual term, which is assumed to follow a normal distribution of $\mathbf{e}\sim \mathbf{N}\left(\mathbf{0},{\boldsymbol{\upsigma}}_{\mathbf{e}}^{\mathbf{2}}\mathbf{I}\right)$. In addition, **G** is the standardized relatedness matrix calculated by GEMMA v0.98.1 software [34] using all SNPs according to [35]:

$$ \mathbf{G}=\frac{\boldsymbol{M}{\boldsymbol{M}}^T}{2{\sum}_{i=1}^m{p}_i\left(1-{p}_i\right)}, $$

where **M** is a matrix of centered genotypes, and ***p***_***i***_ is the minor allele frequency of **SNP**_**i**_.

### Strategies for selecting the feature markers in genomic prediction

In order to improve the predictive ability of whole genome prediction, four strategies were used to preselect SNPs from the WGS data as genomic feature markers, including 1) SNPs preselection based on the GWAS results (abbreviation as “S_ GWAS”); 2) SNPs preselection based on the genome positions of significant genes from the transcriptome-wide association study (TWAS) (abbreviation as “S_ TWAS”); 3) SNPs preselection based on the results of the eQTL mapping of all genes (abbreviation as “S_eQTL_A”); and 4) SNPs preselection based on the results of the eQTL mapping of significant genes from TWAS (abbreviation as “S_eQTL_S”). In all scenarios, if there was no gene or SNP remained after the cut-off thresholds of different categories, the top two genes or five SNPs were exacted as feature markers.

### SNPs preselection based on the GWAS results (S_GWAS)

In order to link genomic variation with complex traits, GWASs were performed for each sex separately for the analyzed traits in the training population. Univariate tests of association were performed using a mixed model approach implemented in the GEMMA v0.98.1 software [34]. The model was

$$ \mathbf{y}=\mathbf{Xb}+\mathbf{Zg}+\mathbf{Sa}+\mathbf{e}, $$

where **y** is a vector of the phenotypic values of lines in the training set; **a** is the additive effect of the candidate variants to be tested for association; **S** is a vector of an SNP, and the other terms are defined as above. A Wald test was applied to test the alternative hypotheses of each SNP in the univariate models. After the GWAS analysis, the SNPs associated with related traits were divided into different categories based on *P* values of less than 0.05, 0.001, 0.0001, 0.00001, or 0.000001. Then, the different categories of significant SNPs were extracted from the WGS data as genomic features, respectively.

### SNPs preselection based on the genome position of significant genes from TWAS (S_TWAS)

In order to link the gene expression level with complex traits, TWASs were performed for each sex separately for the analyzed traits in the training population. The univariate tests of association were performed using a mixed model approach implemented in ‘rMVP’, a package in R (https://github.com/xiaolei-lab/rMVP). The model was

$$ \mathbf{y}=\mathbf{Xb}+\boldsymbol{Z}\mathbf{g}+\boldsymbol{T}\mathbf{u}+\mathbf{e}, $$

where **y** is a vector of the phenotypic values of lines in the training set; ***T*** is a vector of a gene expression level of lines in the training set; **u** is the genetic effect of the candidate genes to be tested for association. and the other terms are defined as above. A Wald test was applied to test the alternative hypotheses of each gene in the univariate models. After the TWAS analysis, the significant gene expression levels associated with related traits were divided into different categories based on *P* values of less than 0.05, 0.001, 0.0001, 0.00001, or 0.000001. Then, the SNPs located in significant genes were extracted as feature markers based on their corresponding genomic positions from the WGS data.

### SNPs preselection based on the results of the eQTL mapping of all genes (S_eQTL_A)

In order to link genome variation with the gene expression level, eQTL mapping was performed for each sex separately for each gene expression level using the WGS data. Univariate tests of association were performed using a mixed model approach implemented in the GEMMA v0.98.1 software [34]. The model was

$$ \mathbf{y}=\mathbf{Xb}+\mathbf{Zg}+\mathbf{Sa}+\mathbf{e}, $$

where ***y*** is a vector of each gene expression level of all lines; ***b*** is the fixed effect, including *Wolbachia* infection status and five major polymorphic inversions [In2L(t), In2R(NS), In3R(P), In3R(K), and In3R(Mo)]; **S** is a vector of the SNP, and the other terms are defined as above. A Wald test was applied to test the alternative hypotheses of each SNP in the univariate models. After eQTL mapping, the significant eQTLs of each gene were divided into different categories based on *P* values of less than 0.05, 0.001, 0.0001, 0.00001, or 0.000001. Then, the different categories of significant eQTLs of each gene were extracted as feature markers from the WGS data, respectively. Because polymorphic inversions had a direct impact on gene expression, moreover, for avoiding spurious associations due to adjustment on both eQTL mapping and TWAS, we added five major polymorphic inversions as fixed effect only in eQTL mapping.

### SNPs preselection based on the results of the eQTL mapping of significant genes (S_eQTL_S)

After the TWAS and eQTL mapping analysis, genes and eQTLs were divided into different categories according to the significance threshold as described above. Using different categories of combination, these significant eQTLs of significant genes were extracted as feature markers from the WGS data, respectively.

### Genomic prediction model

The breeding values of the genotyped individuals were estimated via genomic best linear unbiased prediction (GBLUP) [35] and a genomic feature BLUP model (GFBLUP) [36]. The statistical model for the GBLUP approaches is

$$ \mathbf{y}=\boldsymbol{Xb}+\boldsymbol{Zg}+\boldsymbol{e}, $$

where ***y*** is a vector of the phenotypic values; ***b*** is Walachia infection status as a fixed effect; and the other parameters are defined as above.

The GFBLUP model was an extended BLUP including two random genetic effects:

$$ \mathbf{y}=\boldsymbol{Xb}+{\boldsymbol{Z}}_{\mathbf{1}}\boldsymbol{f}+{\boldsymbol{Z}}_{\mathbf{2}}\boldsymbol{r}+\boldsymbol{e}, $$

where ***y*,** ***b*,** ***X,*** and ***e*** are the same as GBLUP, ***f*** is the vector of the genomic values captured by the genetic markers linked to the genomic feature of interest, following a normal distribution of $\boldsymbol{f}\sim \mathbf{N}\left(\mathbf{0},{\boldsymbol{\upsigma}}_{\boldsymbol{f}}^{\mathbf{2}}{\boldsymbol{G}}_{\boldsymbol{f}}\right)$; and ***r*** is a vector of genomic values captured by the remaining set of genetic markers, following a normal distribution of $\boldsymbol{r}\sim \mathbf{N}\left(\mathbf{0},{\boldsymbol{\upsigma}}_{\boldsymbol{r}}^{\mathbf{2}}{\boldsymbol{G}}_{\boldsymbol{r}}\right)$. ***Z***_**1**_ and ***Z***_**2**_ are the incidence matrices relating the additive genetic values (***g*** and ***f***) to the phenotypic records**.** ***G***_***f***_ and ***G***_***r***_ were constructed according to [35] using the preselected and remaining markers, respectively.

In this study, the variance components were estimated in the training set using the REML algorithm via the LDAK software [33]. Finally, using the dispersion matrices as define in [37] and the variance components, predictions of genetic values of testing sets were obtained by solving the mixed model equations.

### Predictive ability evaluation

The Pearson’s correlation and regression coefficients between the predicted genetic values and the true phenotypic values were used to assess the accuracy and the bias of genomic prediction. The true phenotypic values represent the fixed effects of original phenotypic observations were corrected. Ten replicates of five-fold cross-validation were used to avoid the uncertainty of predictive correlations in this study. Briefly, the genotyped individuals were randomly divided into five subsets. One subset was selected as the validation set, and the remaining four were used as the reference set. Then the cross-validation process was repeated five times to ensure that each subset was validated once. Finally, the average accuracy values and the bias of genomic prediction for the ten replicates of five-fold cross-validation were reported.

## Results

### Summary statistics and genetic parameter estimations of the analyzed traits

Before performing genomic prediction, the summary of statistics and genetic parameter estimations for the traits were performed in the male and female lines, and the detailed results were shown in Table 1. The results showed that the times of the startle response in the female lines (average 28.68 s; range: 14.13–41.25) were similar to those in the male lines (average 28.25 s; range: 13.38–42.10). However, the times of starvation resistance in the female lines (average 60.43 h; range: 34.45–106.56) were much longer than those in the male lines (average 45.52 h; range: 21.28–72.00). The standard deviations were 6.37 and 6.45 for the startle response and 12.61 and 9.40 for starvation resistance in the female and male lines, respectively. The coefficients of variation were 22.21% and 22.83% for the startle response and 20.87% and 20.65% for starvation resistance in the male and female lines, respectively. This indicated that substantial phenotypic variation exists among these traits. Furthermore, the values (standard error) of the heritability estimates were 0.771 (0.191) and 0.691 (0.222) for the startle response and 0.999 (0.083) and 0.999 (0.071) for starvation resistance in the male and female lines, respectively, indicating that they are high-heritability traits. Using likelihood ratio tests, the levels of significance of the heritability estimates were 0.003 and 0.011 for the startle response and 0.0002 and 0.00002 for starvation resistance, indicating a significant genetic contribution to phenotypic variability.

**Table 1:** Summary statistics and genetic parameter estimations of the analyzed traits

**Traits**	**Startle response, s**		**Starvation resistance, h**	
**Sex**	**Female**	**Male**	**Female**	**Male**
N^a^	199	199	198	198
Min	14.13	13.38	34.45	21.28
Max	41.25	42.10	106.56	72.00
Mean	28.68	28.25	60.43	45.52
SD^b^	6.37	6.45	12.61	9.40
CV^c^	22.21%	22.83%	20.87%	20.65%
Heritability(SE^d^)	0.771 (0.191)	0.691 (0.222)	0.999 (0.083)	0.999 (0.071)
Pval of LRT^f^	0.003	0.011	0.0002	0.00002

^a^*N:* Number of individuals; ^b^*SD:* Standard deviation; ^c^*CV* Coefficient of variation; ^d^*SE:* Standard error; ^f^Pval of LRT: *P*-value obtained from likelihood ratio test

### SNPs preselection based on the GWAS results (S_GWAS) with different *P*-value cutoffs for genomic prediction

Using S_GWAS with different *P*-value cutoffs, the accuracy values of both GBLUP and GFBLUP were shown in Table 2. When GBLUP was performed using the complete WGS data, the prediction accuracy values were 0.208 ± 0.020 and 0.181 ± 0.022 for the startle response and 0.272 ± 0.017 and 0.307 ± 0.015 for starvation resistance in the female and male lines, respectively (Table 2). Using S_GWAS with the optimal *P*-value cutoffs (*P* <  0.05), the accuracy values of GBLUP were 0.186 ± 0.021 and 0.158 ± 0.022 for the startle response and 0.207 ± 0.020 and 0.268 ± 0.020 for starvation resistance in the female and male lines, respectively (Table 2). These accuracy values, however, were still lower than those of GBLUP with the complete WGS data. Furthermore, when was using S_GWAS to perform the genomic prediction, the accuracy of GBLUP increased with the *P*-value cutoffs (Table 2). In other words, the accuracy of GBLUP increased with the number of SNPs (Table S2). For example, the number of SNPs increased from 11 to 100,708, meanwhile, the accuracy of GBLUP increased from 0.066 to 0.186 for Startle Response in the female lines. Using S_GWAS with the optimal *P*-value cutoffs, the accuracy of GFBLUP was much lower than that of GBLUP (Table 2). In addition, there was no obvious trend for the accuracy of GFBLUP using different *P*-value cutoffs to preselect SNPs. Overall, using S_GWAS provided lower accuracy and a larger bias of genomic prediction compared to using the complete WGS data for both GBLUP and GFBULP (Table 2, Table S1).

**Table 2:** Prediction accuracies using SNPs preselection based on GWAS results (S_GWAS)

**Model**	***P*-value cutoffs**^**a**^	**Prediction accuracy (Mean ± SE**^**b**^)			
		**Startle response**		**Starvation resistance**	
		**Female**	**Male**	**Female**	**Male**
GBLUP^d^	All^c^	0.208 ± 0.020	0.181 ± 0.022	0.272 ± 0.017	0.307 ± 0.015
	< 0.05	0.186 ± 0.021	0.158 ± 0.022	0.207 ± 0.020	0.268 ± 0.020
	< 0.001	0.097 ± 0.025	0.087 ± 0.022	0.135 ± 0.025	0.140 ± 0.019
	< 0.0001	0.065 ± 0.018	0.053 ± 0.020	0.100 ± 0.019	0.032 ± 0.021
	< 0.00001	0.066 ± 0.019	0.060 ± 0.025	0.004 ± 0.021	−0.056 ± 0.019
GFBLUP^e^	< 0.05	0.054 ± 0.026	0.049 ± 0.024	0.115 ± 0.025	0.121 ± 0.024
	< 0.001	0.083 ± 0.026	0.034 ± 0.023	0.036 ± 0.025	0.047 ± 0.019
	< 0.0001	0.041 ± 0.020	0.045 ± 0.021	0.130 ± 0.021	0.084 ± 0.020
	< 0.00001	0.068 ± 0.019	0.061 ± 0.025	0.101 ± 0.024	0.045 ± 0.017

^a^*P*-value cutoffs: using different *P*-value cutoffs to preselect SNPs from whole genome sequencing (WGS) data based on the results of genome-wide association study (GWAS); ^b^*SE:* Standard error; ^c^*All*: All SNPs of WGS data; ^d^*GBLUP* Genomic best linear unbiased prediction; ^e^*GFBLUP:* Genomic feature best linear unbiased prediction

### SNPs preselection based on the TWAS results (T_GWAS) with different *P*-value cutoffs for genomic prediction

The accuracy of both GBLUP and GFBLUP using S_TWAS with different *P*-value cutoffs was shown in Table 3. The results showed that the accuracy of GBLUP using S_TWAS with the optimal *P*-value cutoffs (*p* <  0.05) was 0.189 ± 0.022 and 0.118 ± 0.022 for the startle response and 0.128 ± 0.017 and 0.196 ± 0.015 for starvation resistance in the female and male lines, respectively (Table 3). However, compared with the complete WGS data, the accuracy of GBLUP cannot be improved by using S_TWAS. In addition, by using S_TWAS to perform genomic prediction, the accuracy of GBLUP always increased with the *P*-value cutoffs or number of SNPs (Table 3 and Table S4), for example, the number of SNPs increased from 594 to 70,285 the accuracy of GBLUP increased from − 0.001 to 0.189 for Startle Response in the female lines. Compared with the GBLUP with S_TWAS, GFBLUP resulted in higher accuracy and smaller bias of genomic prediction, except for the startle response using *P*-value cutoffs less than 0.05 (Table 3 and Table S3). But these accuracies still did not higher than using the complete WGS data in GBLUP. However, by using *P*-value cutoffs less than 0.0001 to preselect the SNPs, the accuracy of GFBLUP was equal to the accuracy of GBLUP with the complete WGS data (Table 3), but the bias of GFBLUP was smaller than that of GBLUP with the complete WGS data (Table S3).

**Table 3:** Prediction accuracies using SNPs preselection based on TWAS results (S_TWAS)

**Model**	***P*-value cutoffs**^**a**^	**Prediction accuracy (Mean ± SE**^**b**^)			
		**Startle response**		**Starvation resistance**	
		**Female**	**Male**	**Female**	**Male**
GBLUP^d^	All^c^	0.208 ± 0.020	0.181 ± 0.022	0.272 ± 0.017	0.307 ± 0.015
	< 0.05	0.189 ± 0.022	0.118 ± 0.022	0.128 ± 0.017	0.196 ± 0.015
	< 0.001	0.029 ± 0.023	−0.008 ± 0.026	−0.007 ± 0.021	0.081 ± 0.022
	< 0.0001	0.005 ± 0.022	−0.042 ± 0.019	0.002 ± 0.020	0.024 ± 0.017
	< 0.00001	−0.001 ± 0.022	−0.042 ± 0.019	0.002 ± 0.020	0.004 ± 0.020
GFBLUP^e^	< 0.05	0.176 ± 0.024	0.106 ± 0.024	0.196 ± 0.020	0.287 ± 0.017
	< 0.001	0.168 ± 0.022	0.140 ± 0.022	0.266 ± 0.018	0.291 ± 0.016
	< 0.0001	0.170 ± 0.019	0.137 ± 0.024	0.272 ± 0.017	0.288 ± 0.018
	< 0.00001	0.169 ± 0.019	0.137 ± 0.024	0.272 ± 0.017	0.296 ± 0.016

^a^*P*-value cutoffs: using different *P*-value cutoffs to preselect genes based on the results of transcriptome-wide association study (TWAS), then extracted SNPs from whole genome sequencing (WGS) data according corresponding genomic positions of genes; ^b^*SE:* Standard error; ^c^*All:* All SNPs of WGS data; ^d^*GBLUP:* Genomic best linear unbiased prediction; ^e^*GFBLUP:* A genomic feature best linear unbiased prediction

### SNPs preselection based on the eQTL mapping results of all genes (S_eQTL_A) with different *P*-value cutoffs for genomic prediction

The accuracy of both GBLUP and GFBLUP using S_eQTL_A with different *P*-value cutoffs was shown in Table 4. The results showed that the accuracy of GBLUP using S_eQTL_A with the optimal *P*-value cutoffs was 0.243 ± 0.020 and 0.220 ± 0.022 for the startle response and 0.274 ± 0.017 and 0.305 ± 0.015 for starvation resistance in the female and male lines, respectively (Table 4). Compared with GBLUP with S_eQTL_A, GFBLUP resulted in lower prediction accuracy, except for the startle response using *P*-value cutoffs less than 0.001 in the male lines (Table 4). Furthermore, by using S_eQTL_A, the trends of the accuracy and bias of genomic prediction were different for the startle response and starvation resistance. For the startle response, by using S_eQTL_A with the optimal strategy, the best accuracy values were represented by increases of 19.12% and 21.55% for GBLUP and 10.78% and 19.89% for GFBULP in the female and male lines, respectively, compared to GBLUP with the complete WGS data (Table 4). However, the biases of genomic prediction with the optimal preselection SNPs were larger than those of the complete WGS data (Table S5). For starvation resistance, lower accuracy and similar biases of genomic prediction were found in the female and male lines, respectively (Table 4 and Table S5). In addition, when the number of SNPs was sufficiently large, the increased number of SNPs decreased the accuracy of GBLUP (Table 4 and Table S6). For example, the number of SNPs increased from 1,038,728 to 2,023,905, the accuracy of GBLUP decreased from 0.241 to 0.220 for Startle Response in the female lines.

**Table 4:** Prediction accuracies using SNPs preselection based on the results of eQTL mapping of all genes (S_eQTL_A)

**Model**	***P*-value cutoffs**^**a**^	**Prediction accuracy (Mean ± SE**^**b**^)			
		**Startle response**		**Starvation resistance**	
		**Female**	**Male**	**Female**	**Male**
GBLUP^d^	All^c^	0.204 ± 0.021	0.181 ± 0.022	0.272 ± 0.017	0.307 ± 0.015
	< 0.001	0.220 ± 0.020	0.178 ± 0.023	0.268 ± 0.017	0.296 ± 0.015
	< 0.0001	0.243 ± 0.020	0.191 ± 0.023	0.278 ± 0.017	0.305 ± 0.015
	< 0.00001	0.241 ± 0.020	0.215 ± 0.022	0.274 ± 0.017	0.300 ± 0.014
	< 0.000001	0.208 ± 0.020	0.220 ± 0.022	0.238 ± 0.018	0.288 ± 0.016
GFBLUP^e^	< 0.001	0.183 ± 0.023	0.181 ± 0.022	0.265 ± 0.017	0.292 ± 0.016
	< 0.0001	0.216 ± 0.021	0.178 ± 0.025	0.265 ± 0.018	0.294 ± 0.015
	< 0.00001	0.226 ± 0.021	0.177 ± 0.024	0.252 ± 0.017	0.276 ± 0.017
	< 0.000001	0.187 ± 0.024	0.217 ± 0.022	0.237 ± 0.02	0.272 ± 0.016

^a^*P*-value cutoffs: using different p-value cutoffs to preselect SNPs from whole genome sequencing (WGS) data based on the results of expression quantitative trait loci (eQTL) mapping of all genes; ^b^*SE:* Standard error, ^c^*All:* All SNPs of WGS data, ^d^*GBLUP:* Genomic best linear unbiased prediction, ^e^*GFBLUP:* A genomic feature best linear unbiased prediction

### SNPs preselection based on the eQTL mapping results of significant genes (S_eQTL_S) with different *P*-value cutoffs for genomic prediction

**Fig. 1:**
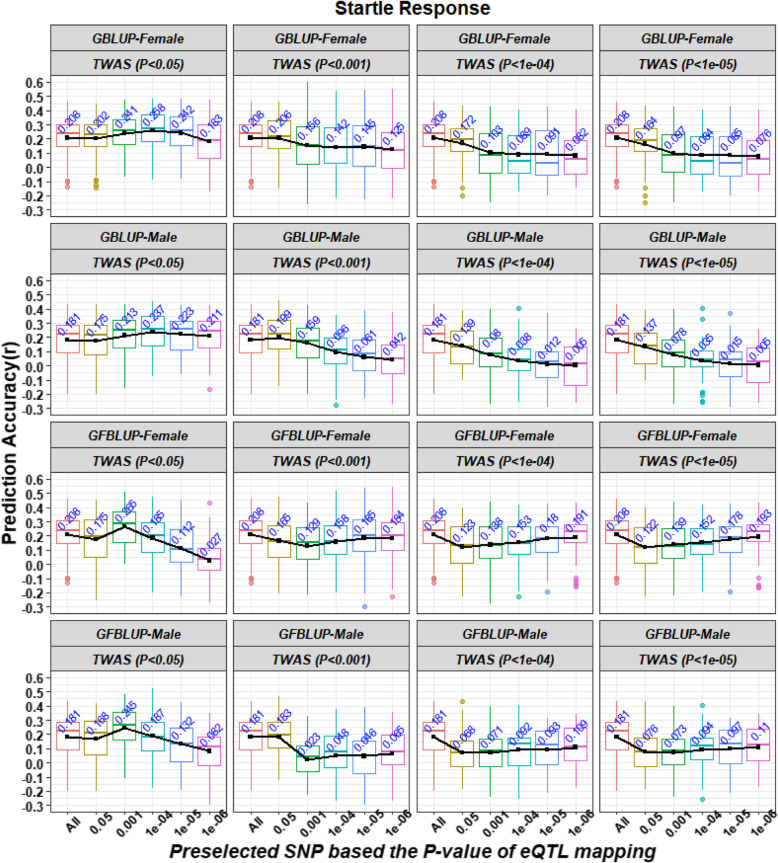
Prediction accuracies of the startle response using S_eQTL_S strategy with different *P*-value cutoffs. S_eQTL_S represents SNPs preselected from WGS data based on the results of the eQTL mapping of significant genes. The Y axis represents the Pearson correlation between the predicted genetic values and the phenotypic values for each trait in the validation sets. Both the X axis and the different colors of box plots represent the SNP datasets preselected from whole genome sequencing data using different *P*-value cutoffs based on the results of the eQTL mapping of significant genes from a transcriptome-wide association study (TWAS). GBLUP-Female and GBLUP-Male refer to performing genomic best linear unbiased prediction (GBLUP) on the female and male lines. GFBLUP-Female and GFBLUP-Male refer to performing genomic feature best linear unbiased prediction (GFBLUP) on the female and male lines. TWAS (*P* < cutoffs) refers to using the *P*-value cutoffs to preselect significant genes from TWAS. Black lines indicate the trend of the average accuracy in different scenarios

**Fig. 2:**
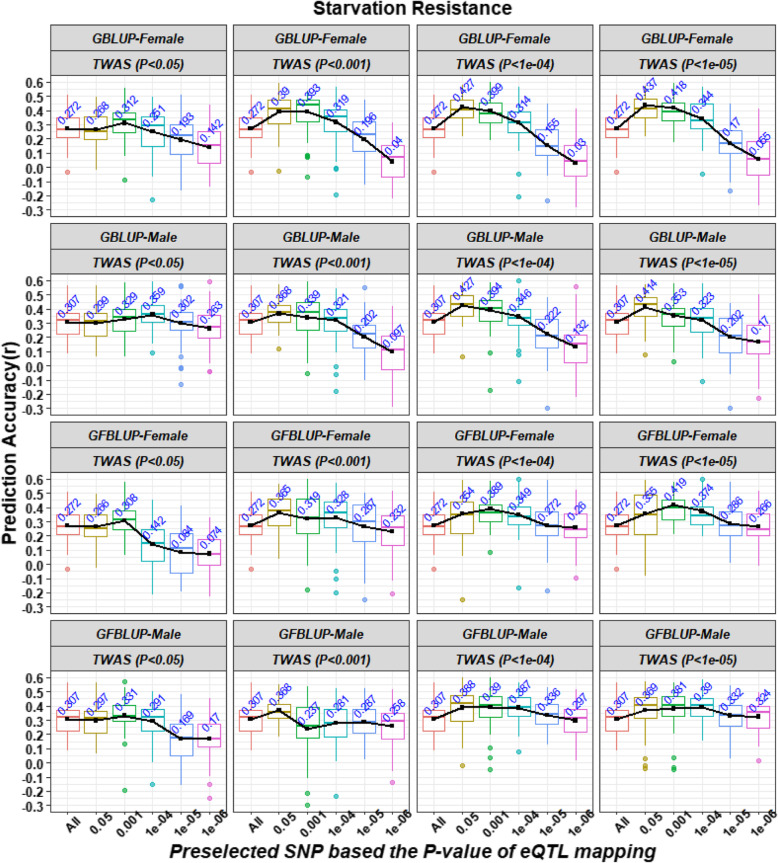
Prediction accuracies of the starvation resistance using S_eQTL_S strategy with different *P*-value cutoffs. S_eQTL_S represents SNPs preselected from WGS data based on the results of the eQTL mapping of significant genes. The Y axis represents the Pearson correlation between the predicted genetic values and the phenotypic values for each trait in the validation sets. Both the X axis and the different colors of box plots represent the SNP datasets preselected from whole genome sequencing data using different *P*-value cutoffs based on the results of the eQTL mapping of significant genes from a transcriptome-wide association study (TWAS). GBLUP-Female and GBLUP-Male refer to performing genomic best linear unbiased prediction (GBLUP) on the female and male lines. GFBLUP-Female and GFBLUP-Male refer to performing genomic feature best linear unbiased prediction (GFBLUP) on the female and male lines. TWAS (*P* < cutoffs) refers to using the *P*-value cutoffs to preselect significant genes from TWAS. Black lines indicate the trend of the average accuracy in different scenarios

The accuracy of genomic prediction for the startle response and starvation resistance using S_eQTL_S with different *P*-value cutoffs is shown in Figs. 1 and 2, respectively. For the startle response, when we used *P*-value cutoffs less than 0.05 or 0.001 to select the significant genes, there existed an appropriate *P*-value cutoff to preselect eQTLs to improve the prediction accuracy of GBLUP and GFBLUP, compared with using GBLUP with the complete WGS data, except when performing GFBLUP on the female lines (Fig. 1). The best accuracy values were 0.258 ± 0.019 and 0.237 ± 0.019 for GBLUP and 0.265 ± 0.018 and 0.245 ± 0.020 for GFBLUP in the female and male lines, respectively (Fig. 1). Compared with the GBLUP using complete WGS data, the accuracy values represented increases of 24.04% and 30.94% for GBLUP and 27.40% and 35.36% for GFBULP in the female and male lines, respectively (Fig. 1). Furthermore, using SNPs preselected with the optimal strategy, the bias of GBLUP was 0.916 ± 0.080 and 0.851 ± 0.079, which are similar to the bias of GBLUP with the complete WGS data in the female (1.113 ± 0.140) and male lines (1.223 ± 0.177), but larger biases of GFBLUP were found in the female (0.415 ± 0.099) and male (0.324 ± 0.096) lines (Table S7). However, when we used *P*-value cutoffs less than 0.0001 or 0.00001 to select the significant genes, we achieved lower accuracy than when using the complete WGS data for both GBLUP and GFBLUP, no matter what *P*-value cutoff was used to preselect eQTLs. For starvation resistance, no matter what *P*-value cutoff was used to preselect significant genes from TWAS results, there always existed an appropriate *P*-value cutoff to preselect eQTLs to improve the accuracy of GBLUP and GFBLUP, compared with GBLUP using complete WGS data (Fig. 2). The best accuracy values were 0.437 ± 0.015 and 0.427 ± 0.015 for GBLUP and 0.419 ± 0.016 and 0.390 ± 0.014 for GFBLUP (Fig. 2). Compared to GBLUP with the complete WGS data, the accuracy values represented increases of 60.66% and 39.09% for GBLUP and 54.04% and 27.04% for GFBULP in the female and male lines, respectively (Fig. 2). Furthermore, by using SNPs preselected with the optimal strategy, the biases of genomic prediction were 0.897 ± 0.064 and 1.217 ± 0.061 for GBLUP and 1.122 ± 0.060 and 1.106 ± 0.062 for GFBLUP in the female and male lines, respectively; these values were similar to or smaller than the biases of GBLUP with the complete WGS data (1.137 ± 0.078 and 1.153 ± 0.065 in the female and male lines, respectively) (Table S6). In addition, the number of SNPs preselected from the WGS data based on the results of the eQTL mapping of significant genes from TWAS are shown in Table S8.

## Discussion

In the present study, we determined the impact of different SNP preselection strategies on prediction accuracy using WGS and gene expression level data. To the best of our knowledge, this is the first time that gene expression level data of whole population were used to preselect feature SNPs to improve the accuracy of genomic prediction. Overall, using the SNPs preselected from WGS data based on gene expression data results in greater accuracy and a smaller bias of genomic prediction for the startle response and starvation resistance in *Drosophila*. Especially in using the SNPs preselected from the eQTL mapping of significant genes, the best accuracy values represented increases of 60.66% and 39.09% for the starvation resistance and 27.40% and 35.36% for startle response in the female and male lines, respectively, compared with GBLUP using the complete WGS data. The new knowledge gained from this study will help scholars enrich the use of omics data to improve the power of genomic prediction.

### Total genomic heritability and prediction accuracy

Before performing genomic prediction, the heritability estimates of analyzed traits were estimated in the male and female lines. We found that the analyzed traits had high heritability, especially for starvation resistance, which almost explains the whole phenotypic variability in both the female and male lines (Table 1). These results are similar to those of a previous study [25] but higher than the results in [26]. This may be due to the quality control of the SNPs, the number of lines, and the line means for phenotypes in the present study, which are the same as those used in [25] and different from those in [16]. The high heritability of the analyzed traits showed that most loci that affect the traits have additive gene actions or contributions from non-additive gene actions at many loci. If additive gene action contributed to high heritability, high heritability would easily achieve a high prediction accuracy [38]. However, in this study, the high heritability of traits did not result in high prediction accuracy. Using WGS data, the accuracy values of GBLUP were 0.208 ± 0.020 (0.181 ± 0.022) for the startle response and 0.272 ± 0.017 (0.307 ± 0.015) for starvation resistance in the female (male) lines (Table 2). One possible reason for this result may be the small size of the reference population for genomic predictions. The other possible reason is that non-additive gene actions might contribute to the high estimated additive genetic variation components [39]. A previous study found that epistasis dominates the genetic architecture of Drosophila’s quantitative traits [40]. Therefore, the high heritability of the analyzed traits was most likely the result of non-additive gene actions. In addition, the accuracy values of GBLUP in the present study were different than those in [5, 17] and similar to those in [25]. This difference may be due to the quality control of SNPs, fixed effects, the cross-validation procedure, or the size of the reference population.

### Genomic feature BLUP model for genomic prediction

GFBLUP is an expansion model for traditional GBLUP that separates the total genomic components into two random genetic components using prior biological knowledge [36]. If a genomic feature contains more causal variants, GFBLUP always has a greater accuracy by adding different weights for the genomic features in the model according to the estimated variance components [17, 36]. Similar results were also found in this study (Fig. 1 and Fig. 2). Furthermore, the accuracy of GFBLUP was influenced by the composition’s genomic features. If the proportion of QTNs in preselected genomic feature markers was very few (or even no), the accuracy of GFBLUP will decrease due to excessive consideration on spurious genomic features [41]. Similar results were also found in this study (Table 2). If the proportion of QTNs in preselected genomic feature markers was large, the GFBLUP further increases its prediction accuracy compared to GBLUP with genomic features only or the complete WGS data [17, 36]. For example, when *P*-values of TWAS and eQTL mapping less than 1e-05 and 0.001 were used to preselect 3,500 and 5,377 SNPs in female and male lines as the genomic feature, the accuracy values of GBLUP with the genomic feature were 0.418 and 0.353 for starvation resistance in the female and male lines, respectively; these values are lower than the accuracy of GFBLUP (0.419 and 0.381 for the female and male lines) (Fig. 2). However, if the proportion of QTNs in preselected genomic feature markers was small, GFBLUP resulted in a lower accuracy compared to GBLUP with genomic features only. For example, when the best parameter (the *P*-values of the TWAS and eQTL mapping were less than 1e-05 and 0.05) were used to preselected 177,035 and 227,569 SNPs in female and male lines as the genomic feature, the accuracy values of GBLUP with the genomic feature were 0.437 and 0.414 for starvation resistance in the female and male lines, respectively, which were higher than the accuracy value of GFBLUP (0.355 and 0.369 for the female and male lines) (Fig. 2). Therefore, the strength of GFBLUP is dependent on the preselection strategy for genomic features.

### SNP preselection strategies influencing prediction accuracy

Performing genomic predictions with prior biological knowledge can improve the predictive ability for complex traits [17, 42, 43]. In this study, using the association analysis method, four strategies were proposed to preselect SNPs from WGS data for genomic prediction. We found that using S_GWAS did not improve the prediction accuracy values, especially for *P*-value cutoffs less than 0.001 (Table 2). Similar results were also indicated in previous studies using SNPs preselected from GWAS [8, 11]. The main reason for this result is that overfitting decreases the prediction accuracy. Overfitting means that a small proportion of variants captured a large proportion of variant components in the prediction model (Table S9 and Table S10). In addition, a smaller number of SNPs were preselected based on the *P*-value of GWAS (Table S2), which is similar to the results of a previous study, which showed that the accuracy of GBLUP decreased with the number of SNPs [5].

Moreover, using S_TWAS with different *P*-value cutoffs to perform genomic prediction resulted in lower prediction accuracy values compared to GBLUP with the complete WGS data (Table 3). However, compared with S_GWAS, there are no overfitting problems in the prediction model using S_TWAS (Table S11 and Table S12). The main factor for the decrease in prediction accuracy is that very few causal variants were detected using the genome position of the significant genes from TWAS (Table S4), as the gene expression level is not only affected by the variants near the regions of this gene (cis-eQTL) but also by the other SNPs in the genome (trans-eQTL) [30]. This phenomenon was confirmed by the greater accuracy values obtained using the SNPs preselected from the eQTL mapping of significant genes (Fig. 1 and Fig. 2).

Furthermore, when using S_eQTL_A with different *P*-value cutoffs to perform genomic prediction, only the startle response produced greater accuracy values compared to GBLUP with the complete WGS data. This is most likely because extreme noise was avoided using eQTL mapping to preselect the SNPs for genomic prediction. Because the expression of numerous genes was found in *Drosophila* [30], combining the significant eQTLs of each gene together almost covered the whole genome (Table S6).

Finally, we combined the strength of TWAS and eQTL mapping by using S_eQTL_S to perform genomic prediction and obtained a higher accuracy and smaller bias of genomic prediction (Fig. 1, Fig. 2 and Table S7), as the link between genomic variation and organismal phenotypes could only be determined by TWAS and eQTL mapping using gene expression data [21]. Briefly, the significant genes from TWAS in the training population represented the main gene expression level directly associated with the traits, and eQTL mapping of the whole population determined the significant SNPs associated with the gene expression level. In addition, combining the analyses of genomic variation with those of transcriptional variation and organismal phenotype variation allowed us to determine the gene networks associated with complex traits [30] so that the gene–gene interactions (epistasis) associated with complex traits could be captured. Overall, using genomic features preselected from multi-omics data is a feasible strategy to improve the power of genomic prediction.

### Challenges for integrating transcriptomic data into genomic predictions

Both this study and several previous studies have indicated that integrating transcriptomic data into genomic prediction is a feasible method to improve the power of genomic prediction [21, 24, 25]. However, using transcriptomic data for genomic prediction in animal and plant breeding remains challenging, because it’s too expensive to perform RNA sequencing for thousands of individuals in routine implementation, especially in practical breeding. Furthermore, unlike SNP, the level of gene expression is tissue-specific and time-dependent. Hence, the RNA must be extracted from the tissue associated with the trait of interest during the correct periods. However, this is very difficult to achieve in practice. In this study, RNA was extracted from whole flies, which ignored the tissue-specific and time-dependent effect such that the gene expression levels represented the average across all tissues [30]. It is important to balance the costs and benefits of using transcriptomic information when integrating transcriptomic data into genomic predictions for practical implementations.

## Conclusion

Overall, multi-omics data can assist genomic feature preselection and improve the performance of genomic prediction. The new knowledge gained from this study will enrich the use of multi-omics in genomic prediction.

## Supplementary information

**Additional file 1: Table S1**. The bias values of genomic prediction using preselected SNPs based on GWAS results (S_GWAS). **Table S2**. The number of preselected SNPs based on the GWAS results (S_GWAS). **Table S3**. The bias values of genomic prediction using preselected SNPs based on TWAS results (S_TWAS). **Table S4**. The number of preselected SNPs based on the TWAS results (S_TWAS). **Table S5**. The bias values of genomic prediction using preselected SNPs based on the results of eQTL mapping of all genes (S_eQTL_A). S6. The number of the preselected SNPs based on the results of eQTL mapping of all genes (S_eQTL_A). **Table S7**. The bias values of genomic prediction using preselected SNPs based on the results of eQTL mapping of significant genes (S_eQTL_S). **Table S8**. The number of preselected SNPs based on the results of eQTL mapping of significant genes (S_eQTL_S). **Table S9**. The variance component of GBLUP using preselected SNPs based on the GWAS results (S_GWAS). **Table S10**. The variance component of GFBLUP using preselected SNPs based on the GWAS results. **Table S11**. The variance component of GBLUP using preselected SNPs based on the TWAS results (S_TWAS). **Table S12**. The variance component of GFBLUP using preselected SNPs based on the TWAS results (S_TWAS).

## Data Availability

The WGS data were downloaded from the *Drosophila* Genetic Reference Panel (DGRP) (http://dgrp.gnets.ncsu.edu/). The mean quantitative trait values and gene expression levels were taken from a previous study [30]. The gene expression data can be found in GEO (accession GSE117850).

## Abbreviations

DGRP: *Drosophila* Genetic Reference Panel
eQTL: Expression quantitative trait locus
G: Standardized relatedness matrix
GBLUP: Genomic best linear unbiased prediction
GFBLUP: Genomic feature best linear unbiased prediction
GS: Genomic selection
GWAS: Genome-wide association study
LD: Linkage disequilibrium
MAF: Minor allele frequency
Omics: Multiple genome-level
QTL: Quantitative trait locus
REML: Restricted maximum likelihood
SNP: Single nucleotide polymorphism
TWAS: Transcriptome-wide association study
WGS: Whole genome sequencing
