# Supplementary material for: Multi-omics-data-assisted genomic feature markers preselection improves the accuracy of genomic prediction
Source: J Anim Sci Biotechnol. 2020 Dec 1;11:109. doi: 10.1186/s40104-020-00515-5 (PMC7708144; doi:10.1186/s40104-020-00515-5)
Supplement: Supplementary file 1 — Additional file 1: Table S1. The bias values of genomic prediction using preselected SNPs based on GWAS results (S_GWAS). Table S2. The number of preselected SNPs based on the GWAS results (S_GWAS). Table S3. The bias values of genomic prediction using preselected SNPs based on TWAS results (S_TWAS). Table S4. The number of preselected SNPs based on the TWAS results (S_TWAS). Table S5. The bias values of genomic prediction using preselected SNPs based on the results of eQTL mapping of all genes (S_eQTL_A). S6. The number of the preselected SNPs based on the results of eQTL mapping of all genes (S_eQTL_A). Table S7. The bias values of genomic prediction using preselected SNPs based on the results of eQTL mapping of significant genes (S_eQTL_S). Table S8. The number of preselected SNPs based on the results of eQTL mapping of significant genes (S_eQTL_S). Table S9. The variance component of GBLUP using preselected SNPs based on the GWAS results (S_GWAS). Table S10. The variance component of GFBLUP using preselected SNPs based on the GWAS results. Table S11. The variance component of GBLUP using preselected SNPs based on the TWAS results (S_TWAS). Table S12. The variance component of GFBLUP using preselected SNPs based on the TWAS results (S_TWAS). [file 40104_2020_515_MOESM1_ESM.docx]

Additional files

**Table S1. The bias values of genomic prediction using preselected SNPs based on GWAS results (S_GWAS)**

| Model | *P*-value cutoffs^1^ | The bias of genomic prediction (Mean±SE^2^) | | | | |  |
| --- | --- | --- | --- | --- | --- | --- | --- |
|  |  | Startle response | |  | Starvation resistance | | |
|  |  | Female | Male |  | Female | Male | |
| GBLUP^4^ | All^3^ | 1.113±0.14 | 1.223±0.177 |  | 1.137±0.078 | 1.153±0.065 | |
|  | < 0.05 | 0.660±0.073 | 0.546±0.077 |  | 0.680±0.068 | 0.920±0.075 | |
|  | < 0.001 | 0.312±0.081 | 0.261±0.072 |  | 0.394±0.070 | 0.379±0.062 | |
|  | < 0.0001 | 0.166±0.047 | 0.126±0.052 |  | 0.288±0.057 | 0.098±0.058 | |
|  | < 0.00001 | 0.149±0.042 | 0.125±0.055 |  | -0.002±0.062 | -0.149±0.045 | |
| GFBLUP^5^ | < 0.05 | 0.087±0.045 | 0.091±0.050 |  | 0.316±0.072 | 0.240±0.042 | |
|  | < 0.001 | 0.143±0.041 | 0.042±0.038 |  | 0.036±0.026 | 0.035±0.014 | |
|  | < 0.0001 | 0.103±0.050 | 0.109±0.052 |  | 0.316±0.057 | 0.215±0.052 | |
|  | < 0.00001 | 0.143±0.038 | 0.129±0.053 |  | 0.235±0.057 | 0.075±0.034 | |

^1^*P*-value cutoffs: using different *P*-value cutoffs to preselect SNPs from whole genome sequencing (WGS) data based on the results of genome-wide association study (GWAS); ^2^SE: standard error; ^3^All: all SNPs of WGS data; ^4^GBLUP: genomic best linear unbiased prediction; ^5^GFBLUP: genomic feature best linear unbiased prediction.

**Table S2. The number of preselected SNPs based on the GWAS results (S_GWAS)**

| *P*-value cutoffs^1^ | Number of preselection SNPs | | | | |
| --- | --- | --- | --- | --- | --- |
|  | Startle response | |  | Starvation resistance | |
|  | Female | Male |  | Female | Male |
| All^2^ | 2,037,712 | 2,037,712 |  | 2,037,712 | 2,037,712 |
| < 0.05 | 100,708 | 101,099 |  | 92,194 | 91,330 |
| < 0.001 | 1,664 | 1,618 |  | 1,663 | 1,443 |
| < 0.0001 | 133 | 123 |  | 159 | 110 |
| < 0.00001 | 11 | 11 |  | 15 | 10 |

^1^*P*-value cutoffs: using different *P*-value cutoffs to preselect SNPs from whole genome sequencing (WGS) data based on the results of genome-wide association study (GWAS); ^2^All: all SNPs of WGS data.

**Table S3. The bias values of genomic prediction using preselected SNPs based on TWAS results (S_TWAS)**

| Model | *P*-value cutoffs^1^ | The bias of genomic prediction (Mean±SE^2^) | | | | |
| --- | --- | --- | --- | --- | --- | --- |
|  |  | Startle response | |  | Starvation resistance | |
|  |  | Female | Male |  | Female | Male |
| GBLUP^4^ | All^3^ | 1.113±0.14 | 1.223±0.177 |  | 1.137±0.078 | 1.153±0.065 |
|  | < 0.05 | 1.017±0.149 | 0.910±0.163 |  | 0.973±0.199 | 0.690±0.066 |
|  | < 0.001 | 3.239±4.82 | -2.059±2.293 |  | 2.167±1.501 | 0.821±0.541 |
|  | < 0.0001 | 3.706±5.264 | -13.976±13.567 |  | 3.408±1.598 | -58.373±62.788 |
|  | < 0.00001 | 3.6±5.265 | -13.976±13.567 |  | 3.408±1.598 | 4.480±3.624 |
| GFBLUP^5^ | < 0.05 | 0.931±0.156 | 0.857±0.191 |  | 1.023±0.071 | 0.732±0.071 |
|  | < 0.001 | 1.064±0.183 | 1.054±0.169 |  | 1.038±0.066 | 1.043±0.079 |
|  | < 0.0001 | 1.058±0.171 | 1.121±0.209 |  | 1.006±0.067 | 1.063±0.072 |
|  | < 0.00001 | 1.052±0.171 | 1.121±0.209 |  | 1.038±0.065 | 1.063±0.072 |

^1^*P*-value cutoffs: using different *P*-value cutoffs to preselect genes based on the results of transcriptome-wide association study (TWAS), then extracted the SNPs from whole genome sequencing (WGS) data according corresponding the genomic positions of genes; ^2^SE: standard error; ^3^All: all SNPs of WGS data; ^4^GBLUP: genomic best linear unbiased prediction; ^5^GFBLUP: a genomic feature best linear unbiased prediction.

**Table S4. The number of preselected SNPs based on the TWAS results (S_TWAS)**

| *P*-value cutoffs^1^ | Number of preselection SNPs | | | | |
| --- | --- | --- | --- | --- | --- |
|  | Startle response | |  | Starvation resistance | |
|  | Female | Male |  | Female | Male |
| All^2^ | 2,037,712 | 2,037,712 |  | 2,037,712 | 2,037,712 |
| < 0.05 | 70,285 | 46,515 |  | 28,268 | 66,676 |
| < 0.001 | 1,473 | 851 |  | 1,073 | 3,970 |
| < 0.0001 | 598 | 457 |  | 850 | 1,470 |
| < 0.00001 | 594 | 457 |  | 850 | 1,259 |

^1^*P*-value cutoffs: using different *P*-value cutoffs to preselect genes based on the results of transcriptome-wide association study (TWAS), then extracted the SNPs from whole genome sequencing (WGS) data according corresponding the genomic positions of genes; ^2^All: all SNPs of WGS data.

**Table S5. The bias values of genomic prediction using preselected SNPs based on the results of eQTL mapping of all genes (S_eQTL_A)**

| Model | *P*-value cutoffs^1^ | The bias of genomic prediction (Mean±SE^2^) | | | | |
| --- | --- | --- | --- | --- | --- | --- |
|  |  | Startle response | |  | Starvation resistance | |
|  |  | Female | Male |  | Female | Male |
| GBLUP^4^ | All^3^ | 1.113±0.14 | 1.223±0.177 |  | 1.137±0.078 | 1.153±0.065 |
|  | < 0.05 | 1.296±0.172 | 1.519±0.229 |  | 1.11±0.077 | 1.100±0.070 |
|  | < 0.001 | 1.344±0.166 | 1.556±0.226 |  | 1.174±0.077 | 1.161±0.068 |
|  | < 0.0001 | 1.374±0.148 | 1.555±0.201 |  | 1.166±0.076 | 1.145±0.069 |
|  | < 0.00001 | 1.341±0.141 | 1.524±0.192 |  | 1.018±0.099 | 1.061±0.067 |
| GFBLUP^5^ | < 0.05 | 1.027±0.141 | 1.256±0.214 |  | 1.094±0.076 | 1.089±0.071 |
|  | < 0.001 | 1.245±0.150 | 1.298±0.206 |  | 1.166±0.083 | 1.131±0.074 |
|  | < 0.0001 | 1.177±0.130 | 1.354±0.174 |  | 1.050±0.076 | 1.055±0.071 |
|  | < 0.00001 | 0.973±0.132 | 1.191±0.171 |  | 0.971±0.075 | 1.011±0.068 |

^1^*P*-value cutoffs: using different *P*-value cutoffs to preselect SNPs from whole genome sequencing (WGS) data based on the results of expression quantitative trait loci (eQTL) mapping of all genes; ^2^SE: standard error; ^3^All: all SNPs of WGS data; ^4^GBLUP: genomic best linear unbiased prediction; ^5^GFBLUP: a genomic feature best linear unbiased prediction.

**Table S6. The number of the preselected SNPs based on the results of eQTL mapping of all genes (S_eQTL_A)**

| *P*-value cutoffs^1^ | Number of preselection SNPs | | | | |
| --- | --- | --- | --- | --- | --- |
|  | Startle response | |  | Starvation resistance | |
|  | Female | Male |  | Female | Male |
| All^2^ | 2,037,712 | 2,037,712 |  | 2,037,712 | 2,037,712 |
| < 0.05 | 2,023,905 | 2,028,614 |  | 2,023,905 | 2,028,614 |
| < 0.001 | 1,800,176 | 1,903,021 |  | 1,800,176 | 1,903,021 |
| < 0.0001 | 1,038,728 | 1,221,627 |  | 1,038,728 | 1,221,627 |
| < 0.00001 | 509,031 | 651,985 |  | 509,031 | 651,985 |

^1^*P*-value cutoffs: using different *P*-value cutoffs to preselect SNPs from whole genome sequencing (WGS) data based on the results of expression quantitative trait loci (eQTL) mapping of all genes; ^2^All: all SNPs of WGS data.

**Table S7.** **The bias values of genomic prediction using preselected SNPs based on the results of eQTL mapping of significant genes (S_eQTL_S)**

| Model | *P*-value cutoffs^1^ (TWAS) | *P*-value cutoffs^1^ (eQTL) | The bias of genomic prediction (Mean±SE^2^) | | | | | |
| --- | --- | --- | --- | --- | --- | --- | --- | --- |
|  |  |  | Startle response | | Starvation resistance | | | |
|  |  |  | Female | Male | | Female | Male | |
| GBLUP^4^ | All^3^ | All^3^ | 1.113±0.140 | 1.223±0.177 | | 1.137±0.078 | | 1.153±0.065 |
|  | < 0.05 | < 0.05 | 1.287±0.171 | 1.506±0.228 | | 0.957±0.135 | | 1.130±0.191 |
|  |  | < 0.001 | 1.050±0.104 | 1.070±0.128 | | 0.992±0.082 | | 0.998±0.094 |
|  |  | < 0.0001 | 0.916±0.080 | 0.851±0.079 | | 0.412±0.070 | | 0.389±0.051 |
|  |  | < 0.00001 | 0.775±0.078 | 0.682±0.064 | | 0.186±0.039 | | 0.243±0.041 |
|  | < 0.001 | < 0.05 | 1.105±0.077 | 1.113±0.068 | | 1.093±0.076 | | 1.103±0.069 |
|  |  | < 0.001 | 1.183±0.073 | 1.204±0.068 | | 1.086±0.066 | | 1.229±0.063 |
|  |  | < 0.0001 | 0.818±0.069 | 1.117±0.063 | | 0.264±0.045 | | 0.804±0.063 |
|  |  | < 0.00001 | 0.477±0.056 | 0.825±0.058 | | 0.134±0.026 | | 0.275±0.046 |
|  | < 0.0001 | < 0.05 | 0.795±0.094 | 0.870±0.110 | | 0.520±0.075 | | 0.590±0.079 |
|  |  | < 0.001 | 0.469±0.080 | 0.460±0.070 | | 0.364±0.072 | | 0.051±0.031 |
|  |  | < 0.0001 | 0.449±0.078 | 0.288±0.076 | | 0.533±0.073 | | 0.099±0.060 |
|  |  | < 0.00001 | 0.525±0.104 | 0.213±0.127 | | 0.698±0.088 | | 0.183±0.103 |
|  | < 0.00001 | < 0.05 | 1.180±0.065 | 1.291±0.063 | | 0.897±0.064 | | 1.217±0.061 |
|  |  | < 0.001 | 0.914±0.060 | 0.794±0.066 | | 0.648±0.071 | | 0.470±0.053 |
|  |  | < 0.0001 | 0.997±0.104 | 0.739±0.059 | | 0.858±0.077 | | 0.574±0.062 |
|  |  | < 0.00001 | 1.797±0.487 | 0.805±0.131 | | 0.903±0.098 | | 0.776±0.058 |
| GFBLUP^5^ | < 0.05 | < 0.05 | 0.612±0.076 | 0.51±0.0780 | | 0.381±0.071 | | 0.198±0.050 |
|  |  | < 0.001 | 0.415±0.099 | 0.324±0.096 | | 0.541±0.091 | | 0.303±0.093 |
|  |  | < 0.0001 | 0.477±0.125 | 0.232±0.129 | | 0.659±0.088 | | 0.497±0.120 |
|  |  | < 0.00001 | 0.565±0.212 | 0.369±0.284 | | 0.856±0.102 | | 0.594±0.126 |
|  | < 0.001 | < 0.05 | 1.070±0.053 | 1.175±0.062 | | 0.766±0.064 | | 0.938±0.061 |
|  |  | < 0.001 | 1.162±0.066 | 0.904±0.060 | | 0.991±0.059 | | 0.878±0.062 |
|  |  | < 0.0001 | 1.655±0.155 | 1.082±0.086 | | 1.111±0.065 | | 0.986±0.059 |
|  |  | < 0.00001 | 3.567±0.621 | 1.723±0.245 | | 1.036±0.081 | | 1.077±0.060 |
|  | < 0.0001 | < 0.05 | 0.590±0.079 | 0.503±0.077 | | 0.383±0.069 | | 0.220±0.051 |
|  |  | < 0.001 | 0.403±0.100 | 0.320±0.095 | | 0.547±0.091 | | 0.314±0.092 |
|  |  | < 0.0001 | 0.464±0.126 | 0.226±0.129 | | 0.662±0.088 | | 0.508±0.119 |
|  |  | < 0.00001 | 0.559±0.214 | 0.402±0.284 | | 0.863±0.102 | | 0.617±0.125 |
|  | < 0.00001 | < 0.05 | 1.062±0.050 | 1.035±0.054 | | 0.751±0.068 | | 0.869±0.061 |
|  |  | < 0.001 | 1.293±0.070 | 0.948±0.073 | | 1.122±0.060 | | 0.969±0.065 |
|  |  | < 0.0001 | 2.047±0.167 | 1.312±0.126 | | 1.240±0.070 | | 1.106±0.062 |
|  |  | < 0.00001 | 5.086±0.906 | 2.350±0.337 | | 1.117±0.077 | | 1.139±0.064 |

^1^*P*-value cutoffs: using different *P*-value cutoffs to preselect SNPs from whole genome sequencing (WGS) data based on the results of expression quantitative trait loci (eQTL) mapping of significant genes from transcriptome-wide association study (TWAS); ^2^SE: standard error; ^3^All: all SNPs of WGS data; ^4^GBLUP: genomic best linear unbiased prediction; ^5^GFBLUP: a genomic feature best linear unbiased prediction.

**Table S8. The number of** **preselected SNPs based on the results of eQTL mapping of significant genes (S_eQTL_S)**

| *P*-value cutoffs^1^ (TWAS) | *P*-value cutoffs^1^ (eQTL) | Number of preselection SNPs | | | |
| --- | --- | --- | --- | --- | --- |
|  |  | Startle response | | Starvation resistance | |
|  |  | Female | Female | Female | Female |
| All^2^ | All^3^ | 2,037,712 | 2,037,712 | 2,037,712 | 2,037,712 |
| < 0.05 | < 0.05 | 2,035,442 | 2,035,255 | 2,033,868 | 2,034,597 |
|  | < 0.001 | 1,377,887 | 1,586,899 | 1,109,765 | 1,502,949 |
|  | < 0.0001 | 482,670 | 582,267 | 324,626 | 531,809 |
|  | < 0.00001 | 148,961 | 180,564 | 92,216 | 150,988 |
| < 0.001 | < 0.05 | 840,084 | 1,246,951 | 800,185 | 1,472,449 |
|  | < 0.001 | 56,231 | 72,273 | 31,570 | 115,140 |
|  | < 0.0001 | 10,852 | 12,863 | 4,484 | 19,412 |
|  | < 0.00001 | 2,303 | 2,817 | 887 | 3,653 |
| < 0.0001 | < 0.05 | 283,722 | 268,341 | 244,508 | 542,116 |
|  | < 0.001 | 13,629 | 10,500 | 5,928 | 1,8190 |
|  | < 0.0001 | 2,776 | 1,796 | 673 | 2,285 |
|  | < 0.00001 | 614 | 359 | 105 | 309 |
| < 0.00001 | < 0.05 | 248,108 | 248,345 | 177,035 | 227,569 |
|  | < 0.001 | 11,608 | 9,619 | 3,500 | 5,377 |
|  | < 0.0001 | 2,407 | 1,654 | 303 | 610 |
|  | < 0.00001 | 540 | 331 | 29 | 82 |

^1^*P*-value cutoffs: using different *P*-value cutoffs to preselect SNPs from whole genome sequencing (WGS) data based on the results of expression quantitative trait loci (eQTL) mapping of significant genes from transcriptome-wide association studies (TWAS); ^2^All: all SNPs of WGS data;

**Table S9. The variance component of GBLUP using preselected SNPs based on the GWAS results (S_GWAS)**

| *P*-value cutoffs^1^ | The average variance component in GBLUP among 10 x 5 cross-validation | | | | | | | |
| --- | --- | --- | --- | --- | --- | --- | --- | --- |
|  | Startle response | | | | Starvation resistance | | | |
|  | Female | | Male | | Female | | Male | |
|  | VS^3^ | VE^4^ | VS | VE | VS | VE | VS | VE |
| All^2^ | 43.74 | 13.33 | 37.30 | 18.17 | 269.97 | -5.71 | 150.42 | -3.42 |
| < 0.05 | 9.87 | -1.54 | 10.79 | -1.49 | 31.10 | -7.21 | 16.62 | -3.84 |
| < 0.001 | 6.75 | 0.02 | 7.17 | 0.07 | 27.79 | -1.13 | 14.66 | -0.04 |
| < 0.0001 | 10.55 | 4.34 | 9.96 | 4.57 | 43.55 | 19.22 | 26.42 | 13.44 |
| < 0.00001 | 12.82 | 20.62 | 13.10 | 19.42 | 46.16 | 71.57 | 29.26 | 43.42 |

^1^*P*-value cutoffs: using different *P*-value cutoffs to preselect SNPs from whole genome sequencing (WGS) data based on the results of genome-wide association study (GWAS); ^2^All: all SNPs of WGS data; ^3^VS: the variance component of preselected SNPs; ^4^VE: the residual variance component.

**Table S10. The variance component of GFBLUP using preselected SNPs based on the GWAS results**

| P-value cutoffs^1^ | | The average variance component in GFBLUP among 10 x 5 cross-validation | | | | | | | | | | | |
| --- | --- | --- | --- | --- | --- | --- | --- | --- | --- | --- | --- | --- | --- |
|  |  | Startle response | | | | | | Starvation resistance | | | | | |
|  |  | Female | | | Male | | | Female | | | Male | | |
|  |  | VR^2^ | VS^3^ | VE^4^ | VR | VS | VE | VR | VS | VE | VR | VS | VE |
| < 0.05 | -10.82 | | 14.39 | -0.20 | -11.23 | 14.02 | 0.92 | -25.00 | 35.59 | -3.27 | -14.89 | 19.81 | -0.88 |
| < 0.001 | -4.07 | | 9.30 | 1.85 | -4.33 | 8.74 | 2.43 | -15.70 | 49.26 | 1.20 | -11.65 | 32.75 | 2.02 |
| < 0.0001 | -0.75 | | 10.63 | 5.02 | -1.41 | 10.05 | 5.85 | 17.96 | 43.04 | 1.84 | 11.81 | 27.78 | 1.27 |
| < 0.00001 | 6.97 | | 12.82 | 13.59 | 3.84 | 13.14 | 15.53 | 66.00 | 46.52 | 6.25 | 43.67 | 28.96 | -0.99 |

^1^*P*-value cutoffs: using different *P*-value cutoffs to preselect SNPs from whole genome sequencing (WGS) data based on the results of genome-wide association study (GWAS); ^2^VR: the variance component of remaining SNPs; ^3^VS: the variance component of preselected SNPs; ^4^VE: the residual variance component.

**Table S11. The variance component of GBLUP using preselected SNPs based on the TWAS results (S_TWAS)**

| *P*-value cutoffs^1^ | Variance component in GBLUP | | | | | | | |
| --- | --- | --- | --- | --- | --- | --- | --- | --- |
|  | Startle response | | | | Starvation resistance | | | |
|  | Female | | Male | | Female | | Male | |
|  | VS^3^ | VE^4^ | VS | VE | VS | VE | VS | VE |
| All^2^ | 43.74 | 13.33 | 37.30 | 18.17 | 269.97 | -5.71 | 150.42 | -3.42 |
| < 0.05 | 28.43 | 11.69 | 22.87 | 18.06 | 104.10 | 54.21 | 87.60 | 2.92 |
| < 0.001 | 4.59 | 35.65 | 2.22 | 38.84 | 7.88 | 148.19 | 16.42 | 72.25 |
| < 0.0001 | 3.50 | 36.73 | 1.51 | 39.58 | 6.52 | 149.52 | 4.25 | 84.21 |
| < 0.00001 | 3.41 | 36.82 | 1.51 | 39.58 | 6.52 | 149.52 | 3.39 | 85.09 |

^1^*P*-value cutoffs: using different *P*-value cutoffs to preselect SNPs from whole genome sequencing (WGS) data based on the results of transcriptome-wide association study (TWAS) and genome annotation; ^2^All: all SNPs of WGS data.^3^VS: the variance component of preselected SNPs; ^4^VE: the residual variance component.

**Table S12. The variance component of GFBLUP using preselected SNPs based on the TWAS results (S_TWAS)**

| *P*-value cutoffs^1^ | Variance component in GFBLUP | | | | | | | | | | | | |
| --- | --- | --- | --- | --- | --- | --- | --- | --- | --- | --- | --- | --- | --- |
|  | Startle response | | | | | | Starvation resistance | | | | | | |
|  | Female | | | Male | | | Female | | | Male | | | |
|  | VR^2^ | VS^3^ | VE^4^ | VR | VS | VE | VR | VS | VE | VR | VS | VE | |
| < 0.05 | 3.71 | 24.83 | 11.63 | 1.92 | 21.69 | 17.32 | 157.83 | 6.80 | -5.35 | 91.29 | -0.13 | | -3.16 |
| < 0.001 | 25.20 | 1.84 | 13.01 | 22.87 | 0.71 | 17.36 | 171.79 | -6.43 | -6.48 | 97.25 | -5.01 | | -4.01 |
| < 0.0001 | 24.99 | 1.77 | 13.27 | 23.22 | 0.02 | 17.82 | 173.17 | -6.97 | -7.12 | 97.01 | -4.93 | | -3.80 |
| < 0.00001 | 24.98 | 1.70 | 13.34 | 23.22 | 0.02 | 17.82 | 173.17 | -6.97 | -7.12 | 96.62 | -4.54 | | -3.77 |

^1^*P*-value cutoffs: using different *P*-value cutoffs to preselect SNPs from whole genome sequencing (WGS) data based on the results of transcriptome-wide association study (TWAS) and genome annotation; ^2^VR: the variance component of remaining SNPs; ^3^VS: the variance component of preselected SNPs; ^4^VE: the residual variance component.
